# Virtual reality simulation to enhance advanced trauma life support trainings – a randomized controlled trial

**DOI:** 10.1186/s12909-024-05645-2

**Published:** 2024-06-17

**Authors:** Tanja Birrenbach, Raphael Stuber, Conrad Eric Müller, Paul-Martin Sutter, Wolf E. Hautz, Aristomenis K. Exadaktylos, Martin Müller, Rafael Wespi, Thomas Christian Sauter

**Affiliations:** 1 Department of Emergency Medicine, Inselspital, Bern University Hospital, University of Bern, Bern, Switzerland Freiburgstrasse 16C, CH-3010; 2 ATLS Switzerland, c/o Swiss Surgeons (SGC/SSC), Aarau, Switzerland; 3 Department of Surgery, Spital Oberengadin, Samedan, Switzerland; 4 Graduate School for Health Sciences, University of Bern, Bern, Switzerland

**Keywords:** ATLS, Virtual reality, Trauma management, Medical education, Simulation

## Abstract

**Background:**

Advanced Trauma Life Support (ATLS) is the gold standard of initial assessment of trauma patients and therefore a widely used training program for medical professionals. Practical application of the knowledge taught can be challenging for medical students and inexperienced clinicians. Simulation-based training, including virtual reality (VR), has proven to be a valuable adjunct to real-world experiences in trauma education. Previous studies have demonstrated the effectiveness of VR simulations for surgical and technical skills training. However, there is limited evidence on VR simulation training specifically for trauma education, particularly within the ATLS curriculum. The purpose of this pilot study is to evaluate the feasibility, effectiveness, and acceptance of using a fully immersive VR trauma simulation to prepare medical students for the ATLS course.

**Methods:**

This was a prospective randomised controlled pilot study on a convenience sample of advanced medical students (*n* = 56; intervention group with adjunct training using a commercially available semi-automated trauma VR simulation, *n* = 28, vs control group, *n* = 28) taking part in the ATLS course of the Military Physician Officer School.

Feasibility was assessed by evaluating factors related to technical factors of the VR training (e.g. rate of interruptions and premature termination). Objective and subjective effectiveness was assessed using confidence ratings at four pre-specified points in the curriculum, validated surveys, clinical scenario scores, multiple choice knowledge tests, and ATLS final clinical scenario and course pass rates. Acceptance was measured using validated instruments to assess variables of media use (Technology acceptance, usability, presence and immersion, workload, and user satisfaction).

**Results:**

The feasibility assessment demonstrated that only one premature termination occurred and that all remaining participants in the intervention group correctly stabilised the patient. No significant differences between the two groups in terms of objective effectiveness were observed (*p* = 0.832 and *p* = 0.237 for the pretest and final knowledge test, respectively; *p* = 0.485 for the pass rates for the final clinical scenario on the first attempt; all participants passed the ATLS course). In terms of subjective effectiveness, the authors found significantly improved confidence post-VR intervention (*p* < .001) in providing emergency care using the ATLS principles. Perceived usefulness in the TEI was stated with a mean of 4 (SD 0.8; range 0–5). Overall acceptance and usability of the VR simulation was rated as positive (System Usability Scale total score mean 79.4 (SD 11.3, range 0–100).

**Conclusions:**

The findings of this prospective pilot study indicate the potential of using VR trauma simulations as a feasible and acceptable supplementary tool for the ATLS training course. Where objective effectiveness regarding test and scenario scores remained unchanged, subjective effectiveness demonstrated improvement.

Future research should focus on identifying specific scenarios and domains where VR can outperform or enhance traditional learning methods in trauma simulation.

**Supplementary Information:**

The online version contains supplementary material available at 10.1186/s12909-024-05645-2.

## Background

Advanced Trauma Life Support (ATLS) stands as the cornerstone of medical trauma training. Developed by the American College of Surgeons and its Committee on Trauma, it offers a comprehensive, concise and safe framework for evaluating and treating victims of traumatic events, especially for medical personal who infrequently encounter trauma [1]. The impact of ATLS on participants' knowledge, clinical skills, organizational abilities, and prioritization approaches is substantiated by Level I evidence [2]. However, for medical students and inexperienced physicians, engaging with the course that teaches ATLS and its prerequisites poses challenges, as they usually do not have much experience in the care for severely injured patients. Furthermore, the ATLS trauma principles encompass many practical skills, that are best studied in a practical setting. Simulation-based training is a valuable modality to complement real-world clinical experiences because it enables control over the sequence of tasks offered to learners, provides opportunities to offer support and guidance to learners, prevents unsafe and dangerous situations, and creates tasks that rarely occur in the real world [3]. However, traditionally simulation-based trainings are highly resource-intensive, incurring personal costs, requiring specialized equipment, and necessitating specific locations. Moreover, adapting these trainings to accommodate an increasing number of students proves challenging. Current trauma education curricula often provide limited scenario practice opportunities, and there is a scarcity on training opportunities for undergraduates [3].

To avoid these problems, new methods of simulation-based education using innovative techniques such as virtual reality (VR) have emerged. VR is a technology that immerses the user in an artificial 3D environment with the use of a head-mounted device (VR headset) [4]. The virtual environment offers various modes of interaction, including handheld devices such as controllers or the innovative option of hand-tracking, allowing users to engage with the virtual world using their own hand movements.VR simulations have proven to be a useful and effective tool, mainly for training surgical and technical skills [4–11]. However, VR can also be used to train nontechnical skills [12].

The evidence of VR simulation training for trauma training is still scarce [3]. Furthermore, so far there is no study including VR simulation into the ATLS curriculum or using ATLS course evaluations as outcome.

VR simulation training has been effectively applied in the practicing the ABCDE (airway, breathing, circulation, disability, exposure) approach [13], which is a key concept also in ATLS. A proof of concept study on VR technology in trauma simulation demonstrated that a VR platform can be used to distinguish decision-making skill levels between novice and expert level providers using ATLS and that VR simulation technology is positively received by learners [14]. Recently, semi-autonomous VR trauma-simulations have been developed [15, 16], and proven non-inferior to conventional manikin-based simulation trauma training in medical students [17]. In a recent pilot study, another VR trauma simulator (TVRSim) was able to discern decision-making abilities among trainees with increasing experience with good learner satisfaction [18].

We are aiming to better prepare medical students for the ATLS training course using a fully immersive VR trauma simulation. To this end, we conducted a prospective randomized controlled pilot study. The feasibility of deployment of a VR trauma simulation as adjunct for preparation, objective and subjective effectiveness of the VR simulation for the ATLS training and the acceptance of the VR trauma simulation (usability, simulator sickness, sense of presence and of immersion, workload, user satisfaction, and technology acceptance) in medical students were investigated.

## Methods

### Study design, setting, and ethical approval

This was a prospective randomised controlled pilot study involving medical students. The study was conducted at the University Emergency Department (ED, Universitätsklinik für Notfallmedizin, UKN) at the Inselspital, University Hospital, Bern, Switzerland, and the Military Physician Officer School 41–1/22 and 41–2/22 (Militärarzt Offiziersschule 41–1/22 and 41–2/22) organized by the Swiss army in Moudon, Switzerland. The study took place from 09.05.2022 until 19.10.2022.

This study was classified as a quality evaluation study by the local institutional review board (Kantonale Ethikkommission Bern (Ethics Committee Bern), BASEC-No**:** Req-2022–00425) and thus exempt from full ethical review. Participants were pseudonymised to ensure privacy.

### ATLS course settings

The ATLS program in Switzerland has been developed by the ATLS Switzerland Committee of the Swiss Surgical Society in accordance with the guidelines of the American College of Surgeons [19]. The purpose of this course is to orient the participants to the initial assessment and management of the trauma patient.

The ATLS student course teaches the concepts of primary and secondary patient assessment, identifies management priorities in a trauma situation and provides a clinical and surgical practice to develop the skills required for the initial assessment and management of patients with multiple injuries. The ATLS course is a mandatory part of the medical curriculum for future military physicians in Switzerland. The duration of the ATLS course is 2.5 days, consisting of 9 h theoretical and 12 h of practical training. The course consists of pre- and post-course tests, core content lectures, interactive case presentations, discussions, development of life-saving skills, practical skills stations, and a final performance proficiency evaluation using a clinical scenario. The preparation for an ATLS course consists of self-study of the course manual before commencement of the course [20]. A written knowledge pretest must be passed to enter the course (40 multiple choice questions with 5 answer options each; passing mark 75% correct answers). At the end of the ATLS course, all candidates take a test run in a training clinical scenario. To pass the whole course, a final knowledge test (40 multiple choice questions with 5 answer options each; passing mark 75% correct answers) and the final clinical scenario using simulated patients need to be passed. The final clinical scenario can be repeated once. The training clinical scenario and the final clinical scenario typically consist of a simulated patient (i.e. simulated wounds) who needs to be treated according to ATLS guidelines with the help of a nurse character.

### Participants and eligibility criteria

We recruited a convenience sample of advanced medical students (mainly year 5 or 6 out of a 6-year curriculum) taking part in the ATLS course of the “Militärarzt Offiziersschule 41–1/22 / Military Physician Officer School 41–1/22” in May/June 2022 and the “Militärarzt Offiziersschule 41–2/22 / Military Physician Officer School 41–2/22” in September/October of the Swiss military corps.

Exclusion criteria were refusal to participate in the study, failure to obtain informed consent and participants who had previously been diagnosed with epilepsy.

The study investigators (RS and RW) informed the participants about the study aims, handed out the information form and ensured the absence of contraindications, responded to the participants' questions, and collected their free, informed and expressed written consent.

### Baseline data

Sociodemographic data (gender, age, year of medical studies, need to wear eyeglasses, prior experience with VR), prior training and experience in trauma care (such as completion of Pre-Hospital Trauma Life Support (PHTLS) provider and/or refresher course [21], self-reported hours spent studying the ATLS course manual), as well as confidence in trauma care, and prior experience with VR, were collected in a survey.

### Randomization

After pseudonymization and collection of baseline data, participants were randomized to control or intervention group by a study investigator (RS) using an Excel randomization function.

### Intervention

The intervention consisted of the application of a fully immersive semi-automated VR trauma training scenario (by SimX Inc., San Francisco, California, United States of America) applying the ATLS principals on a polytraumatized virtual patient using a Meta Quest 2 (Meta Platforms, Inc.; Menlo Park, California, United States of America) headset and controllers. Neither company was involved in any aspects of the study. The hardware used for this study consisted of an OMEN laptop 15-dc0xxx by Hewlett-Packard (HP Inc, 1501 Page Mill Road, Palo Alto, California, 94,304, United States of America). The application was controlled by a study investigator (RW), who acted as a moderator, giving the appropriate prerecorded verbal responses, and initiating the appropriate physiological response (automated scenario progression) (Supplemental Materials Figure S1).

The participants in the intervention group completed their training in the VR simulation in coordination with their military obligations in a period of 5–7 days before the ATLS course. They did first undergo a guided 15-min orientation session with a specific task list to familiarize themselves with the VR environment with a peer tutor (RW) available for instructions as needed. Directly afterwards, the participants underwent the study simulation (patient suffering from a blunt polytrauma with tension pneumothorax and major hemorrhage in the pre-hospital setting). The management principles were based on ATLS principles. These included appropriate completion of the primary survey, responding to vital-monitor cues and recognizing life-threatening situations. If the underlying problem in breathing and circulation were not addressed in a timely manner, clinical deterioration (including cardiac arrest) occurred. Similarly, physiologic improvements occurred if correct and timely interventions took place. Typical actions and interventions included moving around in the ambulance and using equipment, attaching patient monitoring, clinical examination including pulse palpation (tactile feedback trough controllers), heart and lung auscultation, ultrasound (eFAST) evaluation (with review of images), placement of intravenous access and tourniquet, performing needle decompression of pneumothorax, placement of airway devices, administration of fluids and medications, or initiation of CPR (with the help of a nurse non-playable character).

In the VR simulation the participant had 45 min to complete the case or retry if case specific urgent actions had not been executed and led to worsening of the patient’s clinical condition. The scenario could be repeated as often as desired. A minimum number of runs was not specified. The training could be ended at any time. The participants' performance according to the ABCDE scheme during the VR simulation was evaluated by one of the study investigators (RW) using a global rating scale.

### Outcome measures

To gain insights into the technical and operational feasibility of VR simulation training, the number of premature terminations, as well as interruptions of the simulation by the participants and the measurement of the time that the participants spend in the virtual environment were recorded. The number of participants who achieved correct stabilisation of the patient and time needed was recorded as a starting point for assessing the difficulty and associated feasibility of the simulation case.

The objective effectiveness of VR simulation was measured using three different approaches. First, all study participants were assessed in a clinical training scenario and a clinical test scenario using the usual ATLS assessment tools with the addition of a subjective global rating scale from 1 to 7 ("The participant correctly applied the ATLS principles"; 1 = strongly disagree to 7 = strongly agree) (comparison between VR and control group). Secondly, the objective effectiveness of the VR trauma simulation was analysed on the results of the pre-test and the final knowledge test, and thirdly, the pass rate of the ATLS course (yes/no) was recorded.

Subjective effectiveness of the VR trauma simulation was measured by the TEI (Training Evaluation Inventory), consisting of 17 statements regarding subjective enjoyment, perceived usefulness, perceived difficulty, subjective knowledge gain, attitudes towards training assessed on a five-point Likert scale (ranging from 1 = totally disagree to 5 = totally agree) [22]. Confidence in applying the ATLS principles was assessed four times during the course of the study: at baseline, after the training (for the VR group only), before, and after ATLS course curriculum (“I feel confident in applying the ATLS principles correctly” (Likert scale from 1 = totally disagree to 5 = totally agree).

The overall acceptance of the VR simulation was evaluated using six established questionnaires, which the participants in the intervention group completed directly after the VR simulation.

Technology acceptance was measured using the fast form of the Technology Acceptance Model (FF-TAM) [23, 24]. 12 items were assessed on a 7-point semantic differential scale (i.e. 1 = inefficient, 7 = efficient).

Usability was assessed using the System Usability Scale (SUS) [25], which is composed of 10 questions with a five-point Likert attitude scale (range 0–100, with an average score of 68; values above 70 are considered “good”); and the After-Scenario Questionnaire (ASQ) [26], which assesses the ease of task completion, satisfaction with completion time and satisfaction with supporting information on a 7-point Likert scale (total score ranges from 1 = full satisfaction to 7 = poor satisfaction).

“Visually-induced motion sickness” was assessed with four-items (The VR training caused nausea/ headache/ blurred vision/ dizziness) taken from the Simulator Sickness Questionnaire (SSQ) from Kennedy et al. (Likert scale from 1 = totally disagree to 5 = totally agree) [27].

Presence and immersion in the virtual world was determined according to the 6-item questionnaire developed by Slater-Usoh-Steed (total score ranges from 1 = no immersion to 7 = full immersion) [28].

Perceived subjective workload on a scale from 0 to 100 was assessed using the National Aeronautics and Space Administration (NASA) Task Load Index [29]. Overstraining is associated with a total score > 60, understraining with a total score of < 37 [30].

The User Satisfaction Evaluation Questionnaire (USEQ) has six questions with a five-point Likert scale to evaluate user satisfaction (total score ranges from 6 = poor satisfaction to 30 = excellent satisfaction) [31].

### Statistical analysis

Data was analysed in Stata® 16.1 (StataCorp, The College Station, Texas, USA).

Baseline characteristics are presented as numbers and percentage or mean (standard deviation, SD) using descriptive statistics as appropriate. Comparisons between two independent groups (e.g. male vs. female; control vs. VR group) were carried out by Chi-square or Wilcoxon rank sum test depending on variable (categorial or continuous).

Pre- and post-simulation comparisons were performed with McNemars test or Wilcoxon signed rank test. Incomplete variables are indicated. No data were imputed. All calculations were intention-to-treat. Only complete data pairs could be evaluated. A *p*-value < 0.05 was considered significant.

## Results

### Sample characteristics

**Fig. 1:**
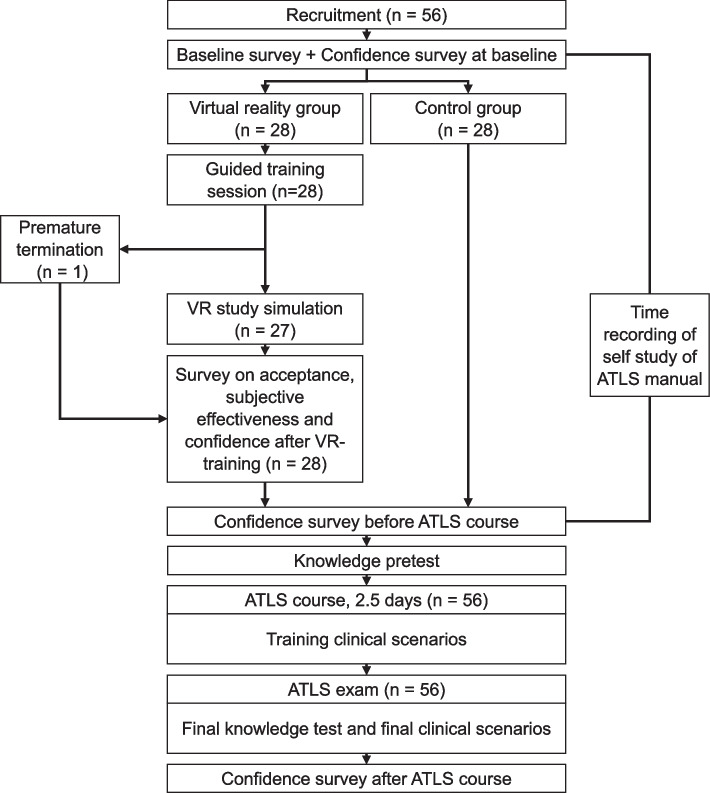
Flowchart flowchart of the study. Abbreviations: ATLS = Advanced Trauma Life Support, VR = Virtual Reality

A total of *n* = 56 students were recruited for the study (*n* = 28 VR group, *n* = 28 control group; total eligible *n* = 58). The flowchart of the study is detailed in Fig. 1. One person did not complete the VR simulation.

No significant differences were found regarding gender, mean age, educational level in medical school, need to wear glasses, previous experience with computer games, or previous experience with VR (Table 1). Likewise, previous education and experience regarding polytrauma care did not show any significant differences. All study participants were PHTLS (Pre-Hospital Trauma Life Support) certified.

**Table 1:** Baseline characteristics

Item	Control group, *n* = 28		VR group, *n* = 28		*p*-value
**Sociodemographic factors**					
Gender, [n (%)]					
male	23	(82.1)	25	(89.3)	.445
Age, in years [mean (SD)]	24.2	(1.6)	24.8	(1.8)	.207
Year medical school, [n (%)]					
Year 5	19	(67.9)	14	(50)	
Year 6	9	(32.1)	14	(50)	.174
Glasses (yes), [n (%)]	16	(57.1)	13	(46.4)	.422
**Previous education and experience in emergency care**					
ATLS Manual self-study hours at baseline, [mean (SD)]	9.1	(6.5)	8.9	(6.4)	.918
PHTLS certified, [n (%)]	28	(100)	28	(100)	
“I have already cared for polytrauma patients according to ATLS”, Likert Scale 1 (totally disagree) to 5 (totally agree), [n (%)]					
1. Totally disagree	20	(71.4)	20	(71.4)	
2. Disagree	5	(17.9)	6	(21.4)	
3. Neutral	2	(7.1)	1	(3.6)	
4. Agree	1	(3.6)	0	(0)	
5. Totally agree	0	(0)	1	(3.6)	.658
**Experience in VR**					
“I regularly play computer games”, Likert Scale 1 (totally disagree) to 5 (totally agree), [n (%)]					
1. Totally disagree	7	(25)	8	(28.6)	
2. Disagree	5	(17.9)	5	(17.9)	
3. Neutral	5	(17.9)	7	(25)	
4. Agree	7	(25)	4	(14.3)	
5. Totally agree	4	(14.3)	4	(14.3)	.875
“I regularly use virtual reality simulations”, Likert Scale 1 (totally disagree) to 5 (totally agree), [n (%)]					
1. Totally disagree	22	(78.6)	24	(85.7)	
2. Disagree	6	(21.4)	3	(10.7)	
3. Neutral	0	(0)	1	(3.6)	
4. Agree	0	(0)	0	(0)	
5. Totally agree	0	(0)	0	(0)	.352

*Abbreviations* *ATLS* Advanced Trauma Life Support, *SD* Standard deviation, *PHTLS* Pre-Hospital Trauma Life Support, *VR* Virtual Reality

## Feasibility and VR simulation details

Table 2 presents the details of the VR simulation. One female participant withdrew from the VR intervention after completing the tutorial and did not partake in the VR simulation. The VR training without the tutorial lasted an average of 33.9 min (SD 7.9), and each participant completed an average of 2.4 trials (SD 0.6). All students were able to stabilize the patient at least once, and this took an average of 19.3 min (SD 8.2).

**Table 2:** VR simulation details

Item	VR group total, *n* = 28	
Premature termination, [n (%)]	1	(3.6)
Interruptions (*n* = 27), [n (%)]	0	(0)
Total trials in VR simulation (*n* = 27), [mean (SD)]	2.4	(0.6)
Total time spend in VR in minutes (*n* = 27), [mean (SD)]	33.9	(7.9)
Correct stabilisation of patient achieved (yes) (*n* = 27), [n (%)]	27	(100)
Time until correct stabilisation of patient in minutes (*n* = 27), [mean (SD)]	19.3	(8.2)
“Student has followed ATLS principles in VR simulation”, global rating scale from 1 (totally disagree) to 7 (totally agree) (*n* = 27), [n (%)]		
1. Totally disagree	1	(3.7)
2. Disagree	2	(7.4)
3. Somewhat disagree	3	(11.1)
4. Neutral	8	(29.6)
5. Somewhat agree	7	(25.9)
6. Agree	4	(14.8)
7. Totally agree	2	(7.4)

*Abbreviations ATLS* Advanced Trauma Life Support, *SD* Standard deviation, *VR* Virtual Reality

## Objective and subjective effectiveness

### Objective effectiveness

The outcomes of the objective effectiveness are displayed in Table 3. There were no significant differences observed between the two groups in terms of their mean scores for the pre- and final multiple choice knowledge tests (*p* = 0.832 and *p* = 0.237 respectively). Similarly, there was no significant difference in the pass rates for the final clinical scenario on the first attempt (*p* = 0.485). All participants ultimately passed the course due to the option to repeat the clinical scenario at the end of the ATLS program.

**Table 3:** Objective effectiveness

Item	Control group, *n* = 28		VR group, *n* = 28		*p*-value
**Knowledge tests**					
Pretest knowledge test, MCQ score in percent compared to the maximum score obtainable, [mean (SD)]	89.2	(7.6)	89.6	(7.4)	.832
Final knowledge test, MCQ score final in percent compared to the maximum score obtainable, [mean (SD)]	90.4	(6.5)	88.3	(6.4)	.237
**ATLS clinical scenarios**					
*Training clinical scenario:*					
Global rating scale “Student has followed ATLS principles”, Likert Scale 1 (totally disagree) to 7 (totally agree), [n (%)]					
1. Totally disagree	0	(0)	0	(0)	
2. Disagree	0	(0)	3	(10.7)	
3. Somewhat disagree	3	(10.7)	6	(21.4)	
4. Neutral	4	(14.3)	3	(10.7)	
5. Somewhat agree	12	(42.9)	2	(7.1)	
6. Agree	5	(17.9)	9	(32.1)	
7. Totally agree	4	(14.3)	5	(17.9)	.028
Passed the training clinical scenario on first attempt, [n (%)]	24	(85.7)	22	(78.6)	.485
*Final clinical scenario:*					
Global rating scale “Student has followed ATLS principles”, Likert Scale 1 (totally disagree) to 7 (totally agree), [n (%)]					
1. Totally disagree	0	(0)	0	(0)	
2. Disagree	0	(0)	3	(10.7)	
3. Somewhat disagree	3	(10.7)	2	(7.1)	
4. Neutral	5	(17.9)	3	(10.7)	
5. Somewhat agree	5	(17.9)	8	(28.6)	
6. Agree	10	(35.7)	6	(21.4)	
7. Totally agree	5	(17.9)	6	(21.4)	.36
Passed the final clinical scenario on first attempt, [n (%)]	26	(92.9)	23	(82.1)	.485
**Overall ATLS course performance**					
Passed the ATLS course (final knowledge test and final clinical scenario, [n (%)]	28	(100)	28	(100)	

*Abbreviations ATLS* Advanced Trauma Life Support, *MCQ* Multiple Choice Question, *PHTLS* Pre-Hospital Trauma Life Support, *SD* Standard deviation, *VR* Virtual Reality

### Subjective effectiveness

**Fig. 2:**
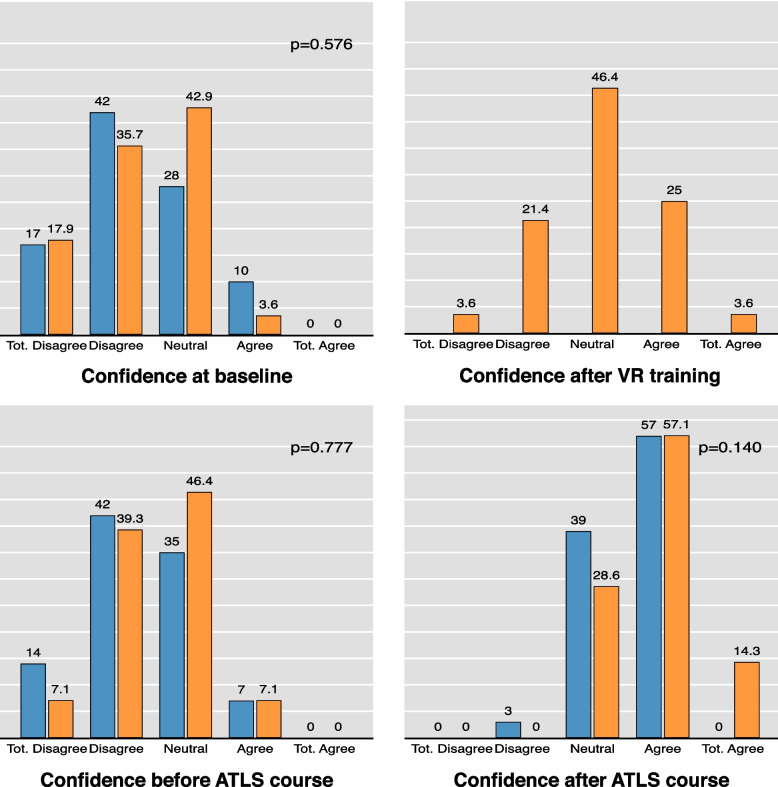
Subjective confidence in treating a polytrauma patient. Confidence in the applying the ATLS principles was assessed at baseline, after the VR training (for the VR group only), before and after ATLS course curriculum (“I feel confident in applying the ATLS principles correctly” (Likert scale from 1 = totally disagree to 5 = totally agree). The blue bars represent the control group and the orange bars the intervention group. Abbreviations: ATLS = Advanced Trauma Life Support, VR = Virtual Reality

With regards to the subjective effectiveness of the VR training, the confidence of the intervention group significantly improved post-VR intervention In the first survey on confidence, 53.6% of the intervention group stated that they totally disagreed or disagreed with the statement "I feel confident in providing emergency care to a polytrauma patient". After the VR training, however, this figure was reduced to 25%. The Training Evaluation Inventory showed good results, particularly for subjective enjoyment (mean 4.6, SD 07). Perceived usefulness was rated with a mean of 4.0 (SD 0.8). Details of the confidence ratings are delineated in Fig. 2, while Table 4 illustrates the results of the Training Evaluation Inventory.

**Table 4:** Training evaluation inventory

Item	VR group, *n* = 28	
TEI, (range 1–5, 5 = good effectiveness), [mean (SD)]		
subjective enjoyment	4.6	(0.7)
perceived usefulness	4	(0.8)
perceived difficulty	4.4	(0.6)
subjective knowledge gain	3.3	(1.0)
attitudes towards training	4.1	(0.7)

*Abbreviations SD* Standard deviation, *TEI* Training Evaluation Inventory, *VR* Virtual Reality

### Acceptance of VR simulation

Results of the dimensions of VR simulation acceptance (usability, simulator sickness, sense of presence and immersion, workload, user satisfaction, and technology acceptance), for the entire sample and distributed by gender are compiled in Table 5. Overall, participants perceived the VR simulation experience positively. Usability as assessed by the SUS was good (mean SUS score 79.4 ± 11.3). The majority of the respondents experienced no to only mild symptoms of visually-induced motion sickness, except for two female participants who reported severe nausea. The average value of immersion according to the questionnaire by Slater-Usoh-Steed was 4.4 ± 1. NASA-Task Load Index total mean score was 49.2 ± 14.7 (range 0–100, 100 = high). User satisfaction was high (USEQ mean score 26.2 ± 3.1 (range 6 = poor satisfaction to 30 = excellent satisfaction). In general, technology acceptance was high (FF-TAM mean value 5.6 ± 0.9; range 1 = poor acceptance to 7 = high acceptance).

**Table 5:** Acceptance of VR simulation by gender

Item	Total, *n* = 28		male, *n* = 25		female, *n* = 3		*p*-value
**Usability**							
SUS total score, (range 0–100, 100 = excellent), [mean (SD)]	79.4	(11.3)	79.2	(11.5)	81.3	(12.1)	.76
ASQ total score, (range 1–7, 1 = full satisfaction), [mean (SD)]	1.9	(1.4)	1.7	(0.9)	4	(2.6)	.003
**Simulator Sickness**							
Visually-induced motion sickness 4-items, Likert Scale 1 (totally disagree) to 5 (totally agree), [n (%)] “The experience caused me…”							
Nausea							
1. Totally disagree	19	(67.9)	19	(76.0)	0	(0)	
2. Disagree	4	(14.3)	3	(12.0)	1	(33.3)	
3. Neutral	1	(3.6)	1	(4.0)	0	(0)	
4. Agree	2	(7.1)	2	(8.0)	0	(0)	
5. Totally agree	2	(7.1)	0	(0)	2	(66.7)	< .001
Headache							
1. Totally disagree	20	(71.4)	18	(72.0)	2	(66.7)	
2. Disagree	4	(14.3)	4	(16.0)	0	(0)	
3. Neutral	2	(7.1)	2	(8.0)	0	(0)	
4. Agree	2	(7.1)	1	(4.0)	1	(33.3)	
5. Totally agree	0	(0)	0	(0)	0	(0)	.266
Blurred vision							
1. Totally disagree	19	(67.9)	17	(68.0)	2	(66.7)	
2. Disagree	7	(25.0)	6	(24.0)	1	(33.3)	
3. Neutral	1	(3.6)	1	(4.0)	0	(0)	
4. Agree	1	(3.6)	1	(4.0)	0	(0)	
5. Totally agree	0	(0)	0	(0)	0	[0]	.954
Dizziness							
1. Totally disagree	18	(64.3)	17	(68.0)	1	(33.3)	
2. Disagree	6	(21.4)	5	(20.0)	1	(33.3)	
3. Neutral	3	(10.7)	3	(12.0)	0	(0)	
4. Agree	0	(0)	0	(0)	0	(0)	
5. Totally agree	1	(3.6)	0	(0)	1	(33.3)	.024
**Sense of presence and immersion**							
Slater-Usoh-Steed total score, (range 1–7, 7 = full immersion), [mean (SD)]	4.4	(1.0)	4.5	(1.0)	3.7	(1.2)	.166
**Workload**							
NASA-Task Load Index total score, (range 0–100, 100 = high), [mean (SD)]	49.2	(14.7)	49.4	(14.2)	48	(22.3)	.883
NASA-Task Load Index subscales (range 0–600, 600 = high), [mean (SD)]							
Mental demand	256.2	(134.0)	255.6	(124.5)	261.7	(237.5)	.943
Physical demand	35.2	(51.3)	39.4	(52.8)	0	(0)	.215
Temporal demand	117.3	(103.7)	115.6	(104.9)	131.7	(113.7)	.805
Performance	127.5	(85.8)	119	(77.9)	198.3	(135.1)	.133
Effort	153.6	(116.0)	166.2	(116.3)	48.3	(30.1)	.097
Frustration	53.2	(65.2)	49.6	(63.4)	83.3	(87.4)	.408
**User satisfaction**							
USEQ total score, (range 6 = poor satisfaction to 30 = excellent satisfaction), [mean (SD)]	26.2	(3.1)	26.5	(3.0)	23.7	(3.1)	.136
**Technology acceptance**							
FF-TAM total score, Likert Scale 1 (no acceptance) to 7 (total acceptance), [mean (SD)]	5.6	(0.9)	5.6	(0.9)	5.7	(0.6)	.847

*Abbreviations ASQ* After Scenario Questionnaire, *ATLS* Advanced Trauma Life Support, *NASA* National Aeronautics and Space Administration, *SD* Standard deviation, *SUS* System Usability Scale, *FF-TAM* Technology Acceptance Model Instrument-Fast Form, *USEQ* User Satisfaction Evaluation Questionnaire

## Discussion

In this prospective randomised controlled pilot study we found the deployment of a VR trauma simulation as adjunct for preparation for the ATLS training course for medical students to be feasible. We did not find any significant differences in objective performance measures after completion of the ATLS course program in the VR group. Subjectively, confidence in treating a polytrauma patient significantly increased after the VR training. The VR trauma simulation was generally well accepted, with good usability and workload, and few VR-associated side-effects.

### Feasibility

Feasibility was confirmed, similar to the findings of Harrington et al. (2018) [14]. The setup using a laptop and stand-alone headset was straightforward, and the commercially available software ran smoothly without technical issues, not causing any interruptions due to technical problems. Participants quickly acclimated to the VR environment and managed the scenarios effectively after a short orientation session. Only one participant had to forgo the simulation due to visually induced motion sickness.

It should be noted that all participants in the intervention group achieved correct stabilisation of the patient. It can therefore be discussed whether the virtual emergency scenario was designed too easy and whether more difficult scenarios need to be programmed in the future.

### Objective outcomes

The utilisation of objective ATLS course completion tests as evaluation markers for ATLS VR training has been demonstrated to be a valuable approach, offering a means of assessing the effectiveness of the training. As our simulation was developed to train practical skills, we did not expect this to influence scores in the multiple choice knowledge test. However, we also did not find a difference in objective outcomes regarding knowledge and solving the clinical scenario between the two groups. As a feasibility study, our study most likely would lack the power to show a small effect to the VR intervention. A large effect, on the other hand, seems unlikely comparing the small VR intervention with the 2.5 days ATLS training course and given, that all participants had previously completed a prehospital trauma training, possibly inducing a ceiling effect. Consequently, it will be the task of further research to evaluate the optimal integration of VR training into an ATLS training curriculum. Further research is necessary to explore the feasibility and effectiveness of incorporating repetitive VR simulations using different scenarios or even partially replacing some resource-intensive ATLS training scenarios with VR.

Our target audience consisted of advanced medical students, who were all PHTLS certified, thus familiar with the ABCDE concept and structured trauma care; however, previous studies suggest that VR training tends to be most beneficial for those in the early stages of their training [18]. While Harrington et al. demonstrated the ability of VR simulations to discern between different levels of expertise, this aspect was not applicable in our study, as we used a homogeneous sample of trainees [14].

### Subjective outcomes

Whereas we found increased confidence after the VR training, we did not find significant differences in confidence after completion of the whole ATLS curriculum. However, given the exhaustive nature of the ATLS curriculum, possible differences induced by adding a 30 min VR simulation are likely to be overcome.

One might however speculate if the VR simulation enhanced motivation and understanding of the participants of preparing for the course and reading the course manual in self-study. One value of the VR simulation could also be to show students their gaps and thus motivate them for the ATLS course or even for self-study of the manual. These effects were not captured in our study.

### Acceptance of VR simulation

The overall evaluation of the VR simulation indicated good usability. This is consistent with previously published studies on VR trauma simulations [14, 16–18]. Most participants found the interface to be simple and intuitive. The majority of respondents reported that the VR simulation was easy to use and did not pose significant difficulties.

Regarding simulator sickness, the majority of participants reported minimal or no symptoms. However, one candidate experienced severe simulator sickness, preventing her from continuing to use the VR simulation after the orientation tutorial. This finding suggests caution should be exercised when considering mandatory VR usage or its application as an assessment method, particularly for older individuals with known motion sickness [16].

The immersion level of the VR simulation was rated as moderate. However, it remains unclear what immersion level is appropriate for medical training or other specific applications. Further investigation is necessary to determine the optimal balance between immersion and usability.

The workload associated with using the VR simulation was comparable to mean scores in a large sample of VR studies [32]. There were no indications of participants being over- or understrained, indicating a good workload balance.

User satisfaction and acceptance were generally high. Participants expressed satisfaction with the VR simulation and had no major concerns. However, it is important to note the potential presence of a novelty effect, as VR technology is still relatively new, and our participants had little or no experience in VR. The novelty effect refers to a boost in the perceived usability of a technology based on its freshness, or the initial improvement in performance when implementing new technology. However, this improvement is not necessarily due to enhanced learning or achievement, but rather a result of heightened interest in the new technology. This effect should be considered when interpreting the results [33].

In terms of usability and simulator sickness, there were statistically significant gender differences in the acceptance of VR simulation. It appears that women are more susceptible to nausea and dizziness in VR simulations, in line with other studies, although the reasons are not perfectly understood [34].

Future applications of VR simulation for trauma training could include remote training and telemedicine, allowing medical professionals to access high-quality trauma training regardless of their geographical location, and continuous education and refresher training. Furthermore, VR simulations can be integrated into assessment processes for trauma training and certification, as automated evaluation methods in conventional trauma trainings are scarce [3]. VR simulations featuring multiplayer options can help to facilitate interdisciplinary training and also enhance communication and leadership skills. VR simulations can be further developed to focus on advanced trauma management skills, such as complex surgical procedures (i.e. resuscitative endovascular balloon occlusion of the aorta (REBOA) or other rare emergency scenarios, that are difficult to simulate in real life due to financial or personal resources or danger to the participants (e.g. mass casualty incidents) [35–37]. Furthermore, other international trauma courses, e.g. the European Trauma Course (ETC), an innovative 2.5 days life support course for training acute care of major trauma patients with a strong focus on team work and practical scenario training, might benefit just as well from adding VR scenario training [38].

Overall, the future applications of VR simulation in trauma training are vast and hold the potential to revolutionize how healthcare professionals are trained, assessed, and prepared to handle trauma cases effectively.

In conclusion, while VR should not be seen as a complete replacement for traditional training, future research endeavors should concentrate on outlining specific scenarios, learner populations and domains where VR surpasses or enhances traditional learning techniques. This approach will facilitate the development of tailored applications, hybrid approaches, and educational innovations that leverage the immersive and experiential benefits of VR to optimize learning outcomes.

### Limitations

These results need to be interpreted with some reservations. First, this was a single tertiary care academic center study with a limited number of participants, that may be affected by selection bias, and thus have impact on generalizability. Since the participants were gathered from a military facility, the demographic composition was predominantly male. Nevertheless, there was an equal distribution between the control and intervention groups. As this was a single-user simulation, it lacks the ability to train team and communication skills, a key in effective trauma team leadership. However, in order to communicate effectively, the team leader must gain pattern recognition, knowledge and confidence which the VR simulation is designed to provide.

One might argue the choice of evaluation methods regarding objective outcomes (medical knowledge test using multiple choice questions, subjective global rating scale for the clinical scenario), however, these are the instruments officially used by the ATLS program.

Transfer to patient outcomes is not addressed in this study, but generally difficult to assess in educational studies.

## Conclusion

This prospective pilot study suggests that the use of VR trauma simulations as an adjunct for the ATLS student course shows potential. While there was no improvement in objective knowledge and practical skills, likely due to limited time for VR intervention combined with weeks of theoretical preparation and a 2.5-day course duration, interesting subjective effects were observed. Participants reported increased confidence in taking care of trauma patients and found the VR training intervention to be usable and acceptable.

It is important to note that while VR simulations have the potential to be a valuable addition to traditional training methods, they should not be viewed as a complete substitute. Future research should therefore focus on identifying the most effective ways to integrate VR training with conventional practical simulation methods, with the aim of achieving the greatest possible benefits for learners.

### Supplementary Information

Supplementary Material 1.

## Data Availability

Data used in this study are available upon reasonable request from the corresponding author at the Emergency Department of the University Hospital Bern, Switzerland to researchers eligible under Swiss legislation to work with codified personal health care data. Eligibility will be determined by Cantonal ethics committee Bern.

## Abbreviations

ASQ: After-Scenario Questionnaire
ATLS: Advanced Trauma Life Support
ED: Emergency department
ETC: European Trauma Course
FF-TAM: Technology Acceptance Model
NASA: National Aeronautics and Space Administration
PHTLS: Pre-hospital Trauma Life Support
REBOA: Resuscitative endovascular balloon occlusion of the aorta
SD: Standard deviation
SUS: System Usability Scale
SSQ: Simulator Sickness Questionnaire
TEI: Training Evaluation Inventory
USEQ: User Satisfaction Evaluation Questionnaire
VR: Virtual reality
