# Supplementary figures and images for: Virtual reality simulation to enhance advanced trauma life support trainings – a randomized controlled trial
Source: BMC Med Educ. 2024 Jun 17;24:666. doi: 10.1186/s12909-024-05645-2 (PMC11184689; doi:10.1186/s12909-024-05645-2)

## Slide 1
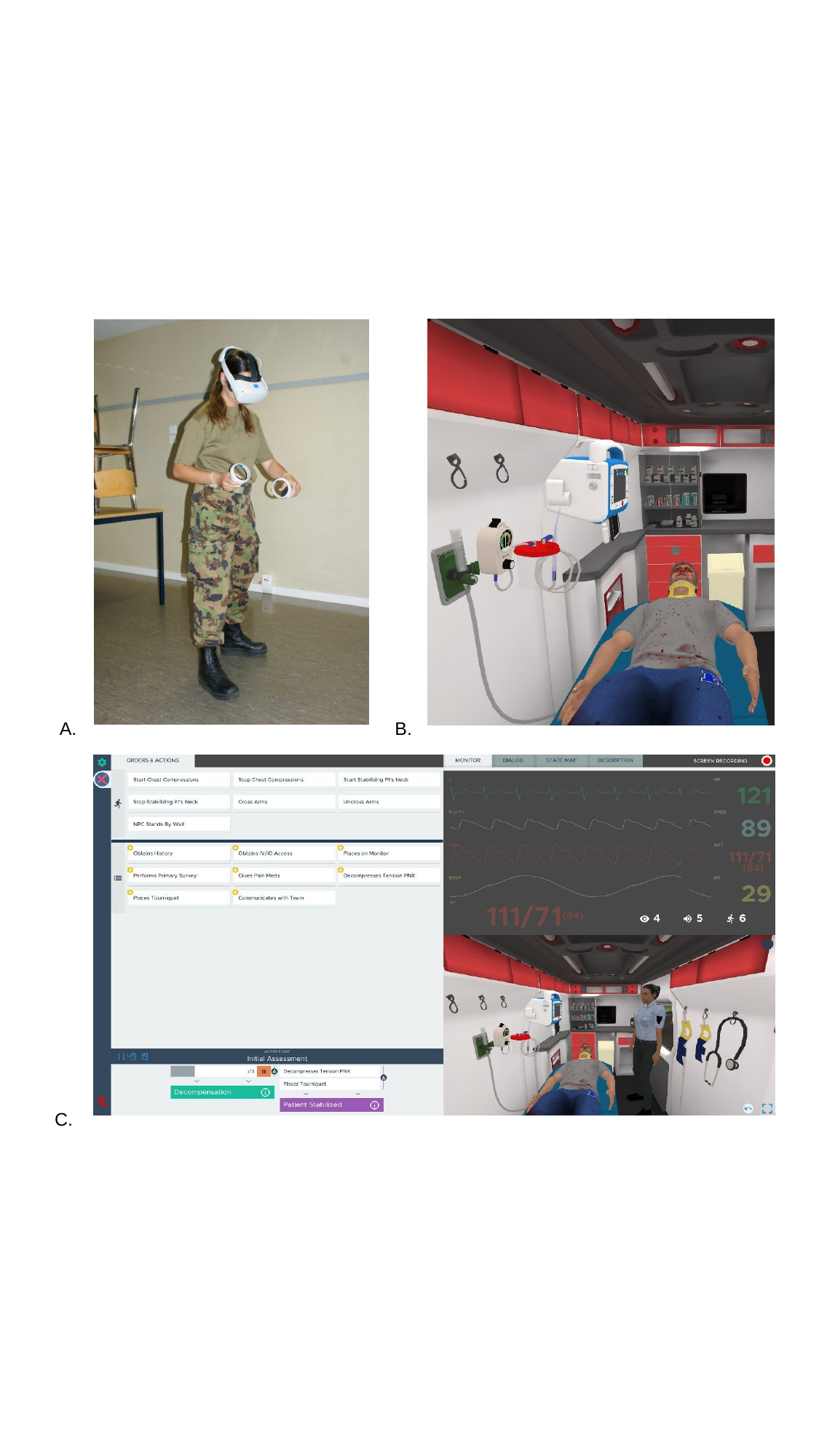

A.
B.
C.

Supplement: Supplementary file 1 — Supplementary Material 1. [file 12909_2024_5645_MOESM1_ESM.pptx]
